# Concurrent Effects of Bleaching Materials and the Size of Root Canal Preparation on Cervical Dentin Microhardness 

**DOI:** 10.22037/iej.v12i3.15774

**Published:** 2017

**Authors:** Maryam Kazemipoor, Shaghayegh Azad, Farnaz Farahat

**Affiliations:** a *Department of Endodontics, Dental School, Shahid Sadoughi University of Medical Sciences, Yazd, Iran; *; b * Department of Operative Dentistry, Dental School, Shahid Sadoughi University of Medical Sciences, Yazd, Iran*

**Keywords:** Hardness Test, Hydrogen Peroxide, Sodium Perborate, Tooth Bleaching

## Abstract

**Introduction:**

The aim of the present study was to evaluate the concurrent effect of root canal preparation size and intra coronal bleaching on dentin microhardness.

**Methods and Materials:**

Seventy-two intact anterior teeth were root canal treated and randomly divided into two groups (*n*=36) according to the size of coronal root canal preparation. The coronal portions of the canals were then enlarged with #2 and 4 Peeso reamers, respectively. Following root canal obturation, teeth were assigned into three groups (*n*=12) to be treated with bleaching agents containing 35% hydrogen peroxide (HP), sodium perborate (SP) and distilled water as control group. The teeth were stored at 37^º^C and 100% humidity for 7 days. Dentinal blocks with 3 mm thickness were obtained from the cervical region and Vickers microhardness number (VHN) were measured for outer and inner dentin in each tooth sample. Data were analyzed using two-way ANOVA and Tukey’s HSD tests.

**Results:**

In the outer dentin, the mean VHN in the HP and control groups showed statistically significant differences (*P*=0.047). The mean VHN of inner dentin for the large preparation size was statistically higher in comparison to the small preparation size (*P*=0.042). There was a statistically significant difference in the mean VHN of inner dentin with small preparation size between HP and SP groups (*P*=0.029) and HP and control groups (*P*=0.021).

**Conclusion:**

Intra coronal bleaching with 35% hydrogen peroxide, affects the inner and outer dentin significantly. Excessive removal of cervical dentin, following root canal preparation, alongside the adverse effect of bleaching materials on dentin could result in the tooth fracture.

## Introduction

Cosmetic dentistry and the growing interest of patients in esthetics is ever evident in modern societies. Treatment options for the discolored teeth include bleaching, laminate, removal of surface stains, microabrasion and placement of porcelain crowns [1]. Bleaching methods due to preservation of tooth structure is a conservative, simple and cost-effective means in the regard [2].

Tooth discolorations have been classified as extrinsic, intrinsic and internalized discoloration [3]. Intrinsic staining in the endodontically treated teeth is a consequence of intra pulpal hemorrhage, pulp tissue remaining, tissue decomposition and presence of sealer material in the pulp chamber [4].

Commercial products commonly used as bleaching agents are based on hydrogen peroxide, carbamide peroxide or sodium perborate [5]. Oxidation reaction of bleaching agents releases free radicals, reactive oxygen molecules and hydrogen peroxide anions which are very reactive and perform their bleaching action through the oxidation of complex chromogenic molecules [6].

Although these agents have therapeutic effect, their application has been associated with some deleterious consequences on the chemical and biomechanical properties of dentin [7]. Alteration in calcium and phosphorus concentration, acid etched appearance (increased roughness and porosity), alteration in the organic and inorganic phases of dentin and hardness reduction are described as dentin modifications following whitening procedure [6, 8].

Since dentinal structure constitutes a major part of root-filled teeth, any alteration in the biomechanical properties of dentin is likely to have an impact on the overall tooth strength [9, 10]. Effect of internal bleaching on the microhardness of endodontically treated root dentin have been evaluated in a limited studies [10-12].

Another concern, related to the structural integrity of the root filled teeth is the final size of root canal preparation and the remaining dentinal thickness [13]. Excessive root canal preparation results in greater susceptibility of tooth to fracture during masticatory loadings [14].

There is a lack of studies investigating whether size of root canal preparation would interfere with microhardness changes of dentin submitted to internal bleaching treatment. Therefore, the aim of the present *in vitro* study was to assess the impact of root canal preparation size on the cervical dentin microhardness following the application of 35% hydrogen peroxide and sodium perborate as intra coronal bleaching agents.

## Materials and Methods

***Selection and preparation of samples***

Seventy-two intact human permanent maxillary and mandibular anterior teeth were chosen for this experimental study. The teeth were thoroughly cleaned and stored in normal saline at 37^º^C to prevent dehydration. 

Conventional access cavities were prepared and remaining pulpal tissue was extirpated using a barbed breach (Dentsply, Maillefer, Ballaigues, Switzerland). Working length was determined by inserting #15 K-file (Dentsply, Maillefer, Ballaigues, Switzerland) into the root canal until the tip was visible at the major apical foramen. The root canals were prepared 1 mm short of this length. 

Biomechanical preparation was carried out using the step-back technique and K-file to a #30 master apical file. After every instrumentation, canals were rinsed with 1 mL freshly prepared 2.5% sodium hypochlorite (NaOCl) solution and a 30-gauge needle (Supa, Tehran, Iran). A final rinse with 10 mL of 17% EDTA solution (Colgate Oral Care Company, Waverley, Australia) followed by 10 mL of 2.5% sodium hypochlorite which was applied for 1 min. 

The teeth were then randomly divided into 2 groups (*n*=36) according to the size of coronal root canal preparation. The coronal portion of the canals were then enlarged to #2 and #4 Peeso reamer (Dentsply, Maillefer, Switzerland), respectively. The root canal was dried with paper points and filled with gutta-percha (PUMADENT, Pumadent Co. LTD, China) and AH-26 (Dentsply Maillefer, Ballaigues, Switzerland) using cold lateral compaction technique. At the cervical third, excessive gutta-percha was removed up to 4 mm below the CEJ using a heated endodontic condenser.

Based on the bleaching agents that were placed into the pulp chamber, samples in each group were randomly assigned into the three groups (*n*=12) as follows: In control groups 1 and 2 (sizes #2 and 4) cotton pellet soaked with distilled water was placed in chamber; in groups 3 and 4 (sizes #2 and 4), cotton pellet soaked with 35% hydrogen peroxide was placed in the chamber; in groups 5 and 6 (sizes #2 and 4), sodium perborate mixed with distilled water to a consistency of wet sand was placed in the chamber. Access cavities were sealed with a temporary restoration (Cavisol, Golchai, Tehran, Iran) and the samples were stored at 37^º^C and 100% humidity for 7 days.

***Microhardness test***

From the CEJ region, dentin specimens with approximately 3.0 mm thickness were prepared using a sectioning machine (Leitz 1600 Microtome, Wetzler, Germany). The sections were grounded using 300-1200 grit silicon carbide paper sequentially. Vickers hardness test (VHT) was performed on the dentin blocks using a microhardness tester (Micromet, Buehler Ltd, Lake Bluff, IL, USA) under 500 g load. Two indentations on the inner and outer dentin were made for each sample. The indentation on the inner and outer dentin was placed approximately 0.5 mm from the root canal space and DEJ respectively. VHN for each indentation was recorded.

**Table 1:** Multiple comparisons using Tukey’s HSD test for microhardness (HP, hydrogen peroxide; SP, sodium perborate

**Size**	**Dentin Location**	**Comparison**	**Mean difference**	***P*** **-value**
**Small**	**Outer**	HP *vs* Control	1.6500	1.000
		SP *vs* Control	5.75833	0.196
		HP *vs* SP	4.10833	0.521
	**Inner**	HP *vs* Control	12.60833	0.021*
		SP *vs* Control	0.58333	1.000
		HP *vs* SP	12.02500	0.029*
**Large**	**Outer**	HP *vs* Control	1.22500	1.000
		SP *vs* Control	8.58333	0.300
		HP *vs* SP	7.35833	0.469
	**Inner**	HP *vs* Control	0.57500	1.000
		SP *vs* Control	3.12500	0.582
		HP *vs* SP	3.70000	0.378

**Figure 1:**
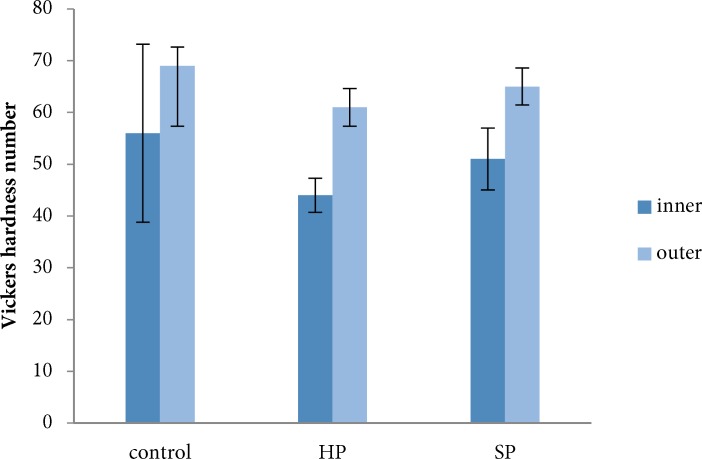
Microhardness of inner and outer dentin with small preparation size after treatment with two bleaching agents HP (hydrogen peroxide), SP (Sodium perborate). Data are presented as mean±SD

**Figure 2:**
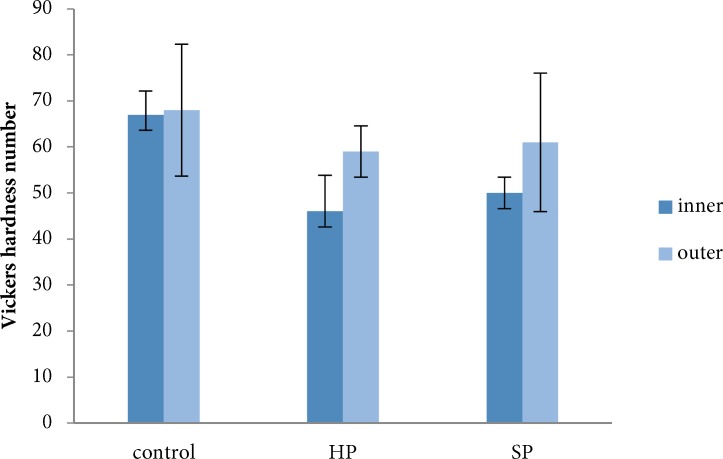
Microhardness of inner and outer dentin with large preparation size after treatment with two bleaching agents HP (hydrogen peroxide), SP (Sodium perborate). Data are presented as mean±SD

Mean and standard deviation were calculated for each experimental group due to normal disturbance of data (according to Shapiro-Wilk test). Determination of the significant differences between groups was rendered through two-way ANOVA and Tukey’s test. The level of statistical significance was assigned at 0.05.

## Results

The mean and standard deviation related to the experimental materials and size of root canal preparation are presented in Figures 1 and 2. The mean VHN of outer dentin was higher than inner dentin for all tested groups.

Considering dentin location, there was significant differences between inner and outer dentin in the HP (*P*=0.0001), SP (*P*=0.0001) and control (*P*=0.008) groups. Also, the groups with two preparation size, showed statistically significant difference between outer and inner dentin (*P*=0.0001). 

Based on ANOVA, in the outer dentin, there was only significant difference related to the bleaching material (*P*=0.044). However, the size and size-material interaction did not show significant differences (*P-*values were 0.664 and 0. 836, respectively). 

The results of multiple comparisons showed statistically significant differences in the outer dentin microhardness only between the HP and control groups (*P*=0.047).

In the inner dentin, there was statistically significant difference with respect to size (*P*=0.027) and size-material interaction (*P*=0.002). The mean VHN of inner dentin for the large preparation size was significantly higher in comparison to the small preparation size (*P*=0.042). There was a statistically significant difference in the mean VHN of inner dentin with small preparation size between HP and SP groups (*P*=0.029) and between HP and control groups (*P*=0.021) (Table 1). 

## Discussion

Internal bleaching is commonly recommended for decolorization of endodontically treated anterior teeth. Although this method results in satisfactory esthetic results, alteration in the chemical and biomechanical (*i.e.* microhardness) properties of dentin must be also expected. Reduction of dentin microhardness has been attributed to the alteration in the water, collagen, noncollagenous and mineral contents of dentin [15, 16].

Because the ultimate strength of a tooth is determined by the dentin substrate, any changes in the quality and quantity of the remaining dentinal structure is likely to have an impact on the overall strength and longevity of the tooth [10]. The concurrent effect of root canal preparation size and the internal bleaching process on the dentin microhardness have not been studied up to the present.

Based on the results of the present study the outer dentin microhardness was significantly higher in comparison with the inner dentin. Tubular density and diameter and the amount of intertubular and intratubular dentin have been associated with the variation of VHN according to dentin location [17, 18].

Since in the present study, bleaching agents were placed in the pulp chamber and not directly on dentin, only diffusion of the bleaching material *via* dentinal tubules would affect the outer dentin microhardness [10].

The mean VHN for outer dentin decreased in the samples with large preparation size that treated with either HP or SP. Most likely, in the presence of patent dentinal tubules, the lower remaining dentinal thickness in the large preparation group would diminish the dentinal bulk with buffering capacity. In this condition, the bleaching agent preserve its oxidizing capacity as outer dentin was approached [19].

In the present study, in the outer dentin there were statistically significant differences only between the HP and control group. It seems that a relatively higher pressure changes following decomposition of HP leads to the more penetration of bleaching material *via* dentinal tubules. Therefore, any changes observed on the outer dentin microhardness are a result of the bleaching material diffusion through the dentinal tubules.

With distance from the pulp chamber, diffusion of the bleaching agents *via* dentinal tubules is limited [10]. This maybe a consequence of lower dentinal tubules density and presence of sclerotic dentin. Since the oxidative effect of peroxide act mainly on the organic component of dentin, intertubular dentin with higher organic content would be more affected compared to the hyper mineralized peritubular dentin [20].

Tubular density, presence of sclerotic dentin, volume of intertubular dentin and its final hardness values after treatment with the bleaching agents would determine the ultimate hardness of the associated dentin.

In the present study, inner dentin microhardness was significantly affected according to size and size-material interaction. The mean VHN of inner dentin for the large preparation size was significantly higher in comparison to the small preparation size. Excessive removal of dentin from root canal walls would expose a dentin with higher microhardness values [17, 18]. Also, there were statistically significant differences in the inner dentin microhardness with small preparation size between the HP-control and HP-SP groups.

Chng *et al. *[10] demonstrated that teeth treated with hydrogen peroxide had lower microhardness than the control group at all dentin locations. In contrast there was no significant difference in microhardness between the control group and teeth treated with either SP mixed with HP or SP mixed with water. These findings are consistent with the result of the current study, although they did not record the root canal preparation size and only implicated that excessive dentin was not removed from the dentinal walls.

Lewinstein *et al.* [11] also recorded that dentin microhardness was significantly reduced after treatment with 30% HP for 5 min, whereas SP mixed with HP for up to 30 min produced no significant alteration in the dentin microhardness.

Many factors could affect the results of the microhardness test. Bleaching material contact time maybe an important factor in the ultimate microhardness of tooth structure [21]. Although, in the clinical conditions satisfactory tooth decolorization is achieved with several-time application of the bleaching material, it is not known whether contact time would cause further dentin weakening. According to Berger *et al.* [22] study, bleaching agents reduced the elastic modules of dentin until 7 days post-bleaching. However after 14 days, the concentration of oxygen was reduced or eliminated which led to a reverse effect of the bleaching material on dentinal mechanical properties. In the present study (similar to the Chng *et al.* [10] survey), samples were kept in contact with bleaching materials for 7 days.

Although sodium perborate paste that prepared with either hydrogen peroxide or distilled water as liquid vehicle has shown similar esthetic results but the water–based paste has lower side effects such as dentin microhardness alteration and external resorption [23].

Since different types of sodium perborate (mono-,tri- or tetra-hydrated) have similar clinical results [24], in the present study we have applied the tetra-hydrated sodium perborate that is commonly used in dentistry.

Sample handling is another factor that could interfere with the results of microhardness tests [25]. Sterilization by autoclaving or chlorine treatment may significantly affect the dentinal hardness [25, 26]. Storage media is also an important factor with regard to dentinal microhardness [12, 15]. Distilled water, because of the lack of calcium and phosphate, has a potential for some dentin demineralization [27]. Also, the storage of samples in 100% relative humidity due to preventing teeth from dehydration, seems to be related to the lower alteration of dental microhardness [8, 28]. In the present study, samples were not autoclaved and stored in isotonic saline and 100% humidity during the experimental period.

Another contributing factor in the effect of bleaching agents on dental structure is the pH of the bleaching material [10]. In this regard, root dentin is more susceptible to demineralization especially in the solutions with a pH lower than 6.7 [29]. Oxidizing ability of HP mainly affects the organic component of dental structure, whereas pH and the solution acidity may affect the mineral components [30, 31]. In the present study, distilled water had a pH of 6.0, 35% hydrogen peroxide had a pH of 1.5 and sodium perborate mixed with distilled water had a pH of 9.5 [31]. 

## Conclusion

Based on the results of the present study, intracoronal bleaching with 35% hydrogen peroxide, effect the inner and outer dentin significantly. The ultimate strength of the endodontically treated teeth is determined with the quantity and quality of the remaining dental structure. Excessive loss of coronal and cervical tooth structure results in greater susceptibility of tooth to fracture during masticatory loadings. Moreover, the deleterious effect of the bleaching agents on the microhardness of inner and outer dentin could impair the tooth resistance to fracture and therefore the excessive loss of cervical dentin structure may tip the balance and lead to tooth fracture during function.
